# Impact of the COVID-19 Pandemic on the Welfare of Animals in Australia

**DOI:** 10.3389/fvets.2020.621843

**Published:** 2021-01-28

**Authors:** Jacqueline Baptista, Dominique Blache, Keren Cox-Witton, Nicola Craddock, Toni Dalziel, Nicolas de Graaff, Jill Fernandes, Ronda Green, Helen Jenkins, Sarah Kahn, Deborah Kelly, Mariko Lauber, Shane K. Maloney, Bridget Peachey, Ian Rodger, Jeremy Skuse, Alan J. Tilbrook, Frederick Rohan Walker, Kelly Wall, Sarah Zito

**Affiliations:** ^1^JB Consulting, Seaforth, NSW, Australia; ^2^School of Agriculture and Environment, The University of Western Australia, Perth, WA, Australia; ^3^The Animal Welfare Collaborative, The University of Queensland, St. Lucia, QLD, Australia; ^4^Wildlife Health Australia, Mosman, NSW, Australia; ^5^Zoo and Aquarium Association, Mosman, NSW, Australia; ^6^Department for Environment and Water, Government of South Australia, Adelaide, SA, Australia; ^7^The Queensland Alliance for Agriculture and Food Innovation, The University of Queensland, St. Lucia, QLD, Australia; ^8^Wildlife Tourism Australia, Running Creek, QLD, Australia; ^9^Animal Health Australia, Turner, ACT, Australia; ^10^Department of Primary Industries and Regional Development, Government of Western Australia, South Perth, WA, Australia; ^11^Greyhound Racing Victoria, West Melbourne, VIC, Australia; ^12^School of Human Sciences, The University of Western Australia, Perth, WA, Australia; ^13^Australian Wool Innovation Limited, Sydney, NSW, Australia; ^14^Department of Agriculture and Fisheries, Queensland Government, Brisbane, QLD, Australia; ^15^Animal Welfare Connections, Hurstbridge, VIC, Australia; ^16^School of Biomedical Sciences and Pharmacy, The University of Newcastle, Newcastle, NSW, Australia; ^17^RSPCA Australia, Deakin West, ACT, Australia

**Keywords:** animal welfare, COVID-19 pandemic, cross-sectoral collaboration, stakeholder networks, Australia

## Abstract

We report on the various responses in Australia during 2020 to minimize negative impacts of the COVID-19 pandemic on the welfare of animals. Most organizations and individuals with animals under their care had emergency preparedness plans in place for various scenarios; however, the restrictions on human movement to contain the spread of COVID-19, coupled with the economic impact and the health effects of COVID-19 on the skilled workforce, constituted a new threat to animal welfare for which there was no blueprint. The spontaneous formation of a national, multisectoral response group on animal welfare, consisting of more than 34 organizations with animals under their care, facilitated information flow during the crisis, which helped to mitigate some of the shocks to different organizations and to ensure continuity of care for animals during the pandemic. We conclude that animal welfare is a shared responsibility, and accordingly, a multisectoral approach to animal welfare during a crisis is required. Our experience demonstrates that to safeguard animal welfare during crises, nations should consider the following: a national risk assessment, clear communication channels, contingency plans for animal welfare, a crisis response group, and support systems for animal care providers. Our findings and recommendations from the Australian context may inform other countries to ensure that animal welfare is not compromised during the course of unpredictable events.

## Introduction

In early 2020, humanity faced an almost unprecedented situation when a novel coronavirus named SARS-CoV-2, causing a disease known as COVID-19, changed life around the world (1). The virus continues to cause death, illness, strain on health systems, societal disruption, and economic damage. Based on experience with other pandemics, like the 1918 influenza pandemic, COVID-19 will likely disrupt human society for years to come (2). The World Health Organization (WHO) described SARS-CoV-2 as “a new strain [of coronavirus] that has not been previously identified in humans” (3). On 30 January 2020 the WHO declared the COVID-19 outbreak to be a “Public Health Emergency of International Concern” (4). On 28 February 2020 the WHO raised COVID-19 to the highest level of global risk (5). Many countries, including Australia, implemented measures that were aimed at reducing the spread of COVID-19, including restrictions on human movement, improved hygiene, and social distancing (henceforth referred to as physical distancing) to reduce human contact.

In Australia, the rapid introduction of government restrictions on human movement posed complex challenges, because Australia is a federation of states and territories (6). On 19 March 2020, the Australian Federal Government closed international borders to all non-citizens and non-residents (7) and then from 26 March 2020, state governments limited travel between all Australian states (8) and to remote communities (9). Meanwhile, on 16 March 2020, the Australian Federal Government imposed physical distancing, and state governments restricted the number of people who could gather in a group, from two to five people, depending on the state (10, 11). Consequently, many public places were closed, including cafes and restaurants, nightclubs, theaters, cinemas, concert halls, zoos, aquariums, and wildlife parks. The containment measures were effective in curtailing new infections, and in June, restrictions were gradually eased (12). A second wave of infections in the state of Victoria in late June prompted the Victorian Premier to place the Melbourne metropolitan area and the Mitchell Shire under tighter restrictions from 7 July 2020 (13). On 2 August 2020, restrictions were expanded to the remainder of the state, and Victoria entered a State of Disaster (14). In response, other jurisdictions tightened their restrictions on the entry of people coming from declared hot spots (15).

Both the health effects of COVID-19 and the measures to contain the pandemic created challenges for the economy, employment, food supply and consumption, and human social activities. Consequently, the pandemic threatened to impact the welfare of animals. Animals are integral to human society. Animals contribute to human well-being, and their welfare often depends on the capacity of humans to provide care for them. In Australia, animal welfare is regulated by legislation in each jurisdiction. The legislation promotes that organizations and individuals with animals under their care plan ahead for the welfare of the animals (16–24). Most organizations with a responsibility for animals have in place emergency preparedness plans for natural disasters and animal disease outbreaks. Nevertheless, these plans were not always suitable for coping with an unpredictable event, such as the COVID-19 pandemic.

A significant risk to animal welfare that defied planning was the extent to which human movement was restricted. The need to restrict human movement and limit social gatherings was identified at the Federal level as early as March, but the manner of implementation of “do not leave home” orders varied between jurisdictions and even local regions. Therefore, multiple organizations made requests to governments for explicit permission for movement associated with animal care (e.g., veterinary care and routine husbandry). Along these lines, the classification of veterinarians, livestock transporters, and animal welfare inspectors as essential services was also pursued by many organizations that are responsible for animal care. In response to these requests, movement restrictions were discussed in a meeting of officials from state, territory and Federal departments on 17 April, and the meeting of Ministers of Agriculture from all jurisdictions on 7 May. Subsequently, the issue of movement restrictions was resolved through clarifications by State and Territory Governments up to the start of the second wave in Victoria. Border restrictions with Victoria were tightened again, causing additional challenges for those moving animals and caring for animals across borders.

In addition to the governmental responses, other sectors of Australian society responded to the COVID-19 pandemic. In the remainder of this article, for each industry and organization that deals with animals and their welfare (hereafter referred to as sectors), we outline the specific effects of the COVID-19 pandemic on the sector and on the welfare of their animals. Where appropriate, we discuss their level of preparedness. Then we describe the spontaneous formation of a national, multisectoral response group, and how this group assisted different animal sectors in dealing with the impacts of the COVID-19 pandemic on animal welfare. We conclude with several recommendations that might be beneficial to countries and groups in safeguarding animal welfare in the course of unpredictable events.

## Sector Responses to The Covid-19 Pandemic

### Red Meat Production

During the early stages of the COVID-19 pandemic there was concern that livestock transport across state and intrastate borders would be restricted and that supply chains would be disrupted, which could impact the welfare of animals in that chain. In Australia, livestock are often transported long distances across borders. In the early weeks of the containment measures, there was panic buying of red meat at supermarkets (25), which put pressure on the supply chain and the demand for transport. There was also concern that livestock saleyards would close because their operation requires the gathering of people. Several organizations that represent the red meat sector sought clarification from authorities, and urged authorities to classify movements that are associated with livestock production as essential services (26). An industry response was coordinated by several peak industry bodies. There were guidelines released around physical distancing in saleyards, and online bidding was encouraged as an alternative to physically attending sales (27). The Australian Federal Government also provided visa extensions for agricultural workers to ensure ongoing support for the husbandry requirements of livestock production (28). Furthermore, in March 2020 the Australian Federal Government introduced the International Freight Assistance Mechanism (IFAM). This was a temporary, emergency measure to help restore critical global supply chains which had been heavily impacted by COVID-19 containment measures around the world. Exports benefitting from this support included lobsters from Western Australia, Victorian lamb, and Tasmanian salmon. IFAM helped to ease pressures on animal production supply chains by maintaining the flow of high-value, time-sensitive, and perishable exports (29).

Concerns were raised about the live export of cattle and sheep, such as getting veterinarians and livestock handlers to the ships and home from international ports. If ship crew contracted the disease, or international borders or ports were closed, contingencies would need to be in place to ensure that the animals health and welfare could be maintained. In the event of canceled shipments, animals would need to be held for longer periods in quarantine feedlots until an alternate destination or domestic processing could be arranged (30).

In late May, the MV *Al Kuwait* livestock carrier arrived in Freemantle Port, Western Australia, with 12 crew members infected with COVID-19 (31). By 2 June, 21 members of the 48-person crew had tested positive to COVID-19, causing the export of 56,000 sheep to the Middle East to be postponed (32). The Australian Federal Government Department of Agriculture, Water and Environment rejected the exporter's initial request for an exemption to the Northern Summer Order, which prohibits live export of sheep by sea to the Middle East during the months of June to mid-September. However, the voyage was later approved to embark subject to a number of conditions; this decision recognized the “exceptional circumstances resulting from the global COVID-19 pandemic” (33).

### Pork and Chicken Meat Production

A major welfare concern for intensive livestock industries, such as pork and chicken meat production, was the risk of disruption to the supply chain if personnel in the supply chain contracted the virus. Concerns included shortages of feed and other supplies, shortages of staff to maintain animal care, and reduced processing capacity, causing overcrowding and a backlog of animals at farms. The latter could lead to the need for the humane destruction of animals on farm. Pigs and broiler chickens were identified as being particularly vulnerable because of their targeted weight gain and the potential for health problems if they are not slaughtered at the scheduled age. Because these production systems operate as a continuous supply of animals, a disruption in slaughter capacity would result in over-production. Broiler chickens reach slaughter age at 30–60 days, so it takes about 2 months to reduce production levels (34). For pigs, the oversupply would last at least 3 months before systems would reach a new steady state at reduced supply, and if the supply was reduced, it would also take several months to return to normal production levels (35).

In the state of Victoria, the second wave of COVID-19 infections in June led to a 34% reduction in meat processing capacity because of measures to avoid the overlap of shifts and to ensure physical distancing (36). When the processing capacity in Victoria was impacted, then the Governments of Victoria and South Australia (SA) arranged for pigs and broilers to be transported across the SA border, and to be processed in SA. The pork industry was relatively well-prepared for the changes in processing capacity, because emergency preparedness plans had been established due to the threat of an incursion of African Swine Fever. Much of the pork industry had strict biosecurity measures in place and had the capacity to isolate animals. Pork processing establishments had the ability to close at short notice. Australian Pork Limited (APL) communicated with pork producers to determine that there would be an extra 2 weeks reserve space on farms to handle the backlog of pigs that would be caused when processing capacity was reduced. APL also investigated the fast tracking of research on humane methods for the mass killing of pigs. In other countries, for example the United States, the closure of meat processing facilities led to hundreds of thousands of pigs and chickens being humanely destroyed (37, 38), but this did not happen in Australia.

### Eggs, Milk, and Wool Production

In livestock sectors where products are harvested on farm, such as in eggs, milk, and wool production, the main concerns during the COVID-19 pandemic were about possible interruptions to the feed supply chain and reduction in workforce capacity. Australian Eggs urged egg farmers to review their access to supplies and to identify any supply chains that might be jeopardized due to national or state restrictions related to the pandemic (39). Representatives from Australian Dairy Farmers, Australian Dairy Products Federation, and Dairy Australia worked to ensure that dairy activities were classified as an “essential service” and implemented measures to keep supply chains operating (40).

In the wool industry, shearers normally travel within Australia, and from New Zealand to Australia, for seasonal shearing and crutching (removal of the perianal wool). The restrictions on international and domestic movement meant that shearers could not travel. Shearing and crutching are done for production purposes but are also critical to the welfare of sheep. Specific welfare risks of not being shorn include build-up of moisture, urine, and feces in the wool, which can lead to fleece rot and flystrike, wool blindness, and skin irritation and infections (41). The wool industry requested visa exemptions for New Zealand shearers and produced guidelines to reduce physical contact during shearing. As of September, the request for exemptions had not been granted.

### Aquaculture

The aquaculture sector was impacted by major market shifts during the COVID-19 pandemic, which were caused by the closure of restaurants and catering services and a reduction of export capacity. At the start of the pandemic, there was some concern that these shifts in market demand could cause financial constraints for producers, who then might not be able to pay for veterinary investigations and feed, which could result in poor health and welfare outcomes (42). Similar to the situation with the pork and chicken supply chain, there was a risk of oversupply in the aquaculture supply chain, which could impact animal welfare. Finished aquaculture stock might not be able to be moved off farm, because aquaculture commonly uses ponds and sea cages that cater for a finite volume of stock.

As the food service and export markets contracted, the aquaculture sector quickly shifted their sales channels to meet the increase in demand by Australian consumers, who began cooking more from home during the COVID-19 pandemic (43). For example, Barramundi farmers urged consumers to buy Australian barramundi (44). By May, international air freight had expanded, partly due to the Australian Federal Government establishing an International Freight Assistance Mechanism (29). With the increased ability of the sector to access international markets, pressure on the supply chain eased.

### Zoos and Aquariums

In the early stages of the pandemic, zoos and aquariums saw a loss of income due to forced closure to the public, which put at risk their ability to provide appropriate care to the exhibited animals. Zoos and aquariums rely on gate entry fees to finance the cost of animal care (e.g., electricity for temperature control and water quality/life support systems, costs of feeding, costs of maintaining animal enclosures, costs of veterinary care and treatment). Without such income, zoos and aquariums had to draw on reserves as well as significantly realign expenditure to sustain appropriate care. Some facilities estimated 8 weeks of resources remained before they would need to permanently close. Normally when a facility closes, animals are relocated to other facilities, but during the COVID-19 pandemic, relocation was not possible because neither the sending nor receiving institution had the financial resources for relocation costs or the care of additional animals. There was concern that pressures to sustain animal care across multiple facilities may lead to difficult decisions. In other countries, the humane destruction of exotic and native animals was being openly considered (45).

Members of the Zoo and Aquarium Association (ZAA) in Australia participate in vital conservation programs, conduct wildlife rescue, and participate in a number of Australian Government Threatened Species Recovery programs. As conservation businesses, they play an important role in the protection and welfare of Australian native species, and a role in rehabilitation and species recovery after the summer bushfire season. Threats to the viability of zoo and aquarium businesses posed by the COVID-19 pandemic put this important work at risk.

When zoos and aquariums were closed to the public, a welfare issue was identified in the reduction of human interaction with the animals. The human-animal relationship is important to the welfare of some captive animals (46), and various zoos and aquariums reported signs of negative affect in their animals during the closure period. For example, Cairns Aquarium observed that larger species, particularly Maori wrasse and Queensland groupers, exhibited depression-like signs, such as refusal to eat (47).

The risk of human-to-animal transmission of SARS-CoV-2 was highlighted by sporadic reports abroad (48). Precautions were taken by zoo and aquarium personnel and veterinarians to minimize contact with animals at high risk, such as large cats and primates. No incidents of SARS-CoV-2 transmission between humans and animals have been reported in Australia.

Given the importance of the sector to tourism, on 28 April, the Australian Federal Government released a $94.6 million package to ZAA accredited zoos and aquariums, and other wildlife businesses, to support the ongoing provision of animal care (49). Non-government zoos and aquariums could apply for the Federal Government JobKeeper program to assist with staffing costs (50). In addition to the support package from the Australian Federal Government, some jurisdictions provided financial support to smaller businesses, with grants administered by state tourism or development agencies (51–53).

Prior to the announcement of the support package from the Australian Federal Government, ZAA had explored animal feed solutions with other animal industries (e.g., the meat processing sector), where collaboration would reduce the cost of care for animals at zoos and aquariums. The use of trucks and provision of whole carcasses at a reduced cost were sought to assist in maintaining animals' care. Following the release of the Australian Federal Government support package, ZAA continued to explore opportunities to reduce the costs of care, because it was recognized that zoos and aquariums might have reduced visitation in the medium term.

### Wildlife Parks and Mobile Exhibitors

Like zoos and aquariums, wildlife parks depend on visitors for revenue. Some wildlife parks did not qualify for grants under the Australian Federal Government support package, but most retained their employees through the Australian Federal Government JobKeeper program. Many wildlife parks received food donations, and others started online crowd funding campaigns (54). As for zoos and aquariums, the animals in wildlife parks probably experienced loss of the enrichment that is normally provided by the interaction with visitors.

Wildlife mobile exhibitors (such as petting zoos and mobile farms) were significantly impacted by the closure of schools and the banning of gatherings such as birthday parties. When public schools re-opened, many exhibitors were still prohibited from entering schools. Because country shows (fairs) can provide up to 70% of the income for some operators, the cancellation of country shows had a significant impact on many wildlife mobile exhibitors. While mobile exhibitors could not access the Federal financial assistance that was provided to the zoo and aquarium sector, some jurisdictions did provide assistance (51, 52). For example, the Queensland Government provided a $0.5 million grant to support the licensed mobile sector. The rationale for that support was that the mobile exhibitor sector in Queensland contributes toward awareness and education of Australia's native species (51). When public schools re-opened, many exhibitors were still prohibited from entering. During this time, the animals in mobile shows were maintained, but many operators faced significant financial issues.

### Wildlife Rescue and Rehabilitation

When the COVID-19 pandemic began, most wildlife rescue organizations were recovering from the impacts of the summer bushfires, which devastated large portions of habitat in eastern and south-eastern Australia (55). Some rescue organizations had financial reserves from bushfire donations. Their ability to collect additional donations, or accept volunteers, was limited, which increased the workload and created the need for alternative feed supplies. Wildlife Health Australia (WHA) was concerned that the COVID-19 crisis could impact animal welfare if workers could not attend to the needs of the wildlife in their care, or free-ranging wildlife under their management (e.g., threatened species programs). WHA highlighted the necessity for people in wildlife care and emergency response roles, both paid and voluntary, to be recognized as “essential workers” by governments (56). WHA also encouraged those who care for wildlife to develop contingency plans in the event that they became sick or had to self-isolate (57). There were also potential impacts on conservation and welfare when wildlife research was discontinued due to public health concerns or financial constraints related to COVID-19.

In some other countries, concern about the potential risk of transmission of COVID-19 from humans to wildlife, particularly bats, resulted in the suspension or restriction of rehabilitation and research (58, 59). Similar concerns were raised in Australia, alongside concerns about the negative welfare impacts if restrictions were imposed. WHA worked with government and non-government stakeholders to assess the risk within the Australian context and to provide advice on biosecurity measures to minimize the risk of transmission while rehabilitation and research continued (60). One positive impact of the COVID-19 pandemic, as a result of the decrease in international travel, may have been a reduction in the lucrative, illegal smuggling of wildlife out of, and into, Australia (61). Reduced traffic through national parks and other wildlife areas likely reduced roadkill and disturbance of animals, but it might have also increased opportunities for smugglers to collect animals undetected.

### Horse and Greyhound Racing

Horse and greyhound racing continued throughout Australia during the pandemic, except in the state of Tasmania (62). Betting continued online, but revenue from on-course betting, gate takings, restaurant and bar services, and stud fees all decreased because of both the economic downturn and the restrictions on attendance at the races. With reduced revenue from racing, there was a concern that horse owners would no longer employ trainers and veterinarians. If trainers were not being paid, they could return horses to their owners, who may not have the facilities, knowledge, or capacity to care for them. The capacity to rescue unwanted horses was also reduced, as horse rescue groups experienced a decrease in donations and were unable to run fundraising events.

Although greyhounds are less expensive to maintain than horses, the greyhound industry was affected financially by the COVID-19 pandemic. Greyhound racing continued under strict COVID-19 containment protocols that were quickly implemented in each jurisdiction, including fewer staff on track, limited participant attendance, the regionalising of race-day officials in some jurisdictions, and the implementation of strict pre-entry health checks. Some jurisdictions also moved to a regional racing format, for a period, where participants were required to race within specific regions. Border closures limited the ability of some greyhound owners and trainers to cross state borders to access greyhound tracks. Despite income loss for some greyhound owners and trainers during this time, care for the animals was maintained.

### Animals Used in Research and Teaching

When the pandemic started, research and teaching institutions responded in the first instance with a change to staffing processes. They divided their animal care staff into two teams, who worked alternate shifts to provide around-the-clock care for animals, as required by Australian laws, and to reduce the risk that an entire workforce would become infected. Access to animal houses was restricted to essential people. Teaching with animals ceased, but most research continued under modified conditions. Animal Ethics Committees (AECs) carefully scrutinized changes to protocols, and the contingency plans that had to be developed for each project. There was a reduction in breeding for animal colonies, where appropriate.

An initial concern was that research animals would have to be humanely destroyed if researchers were unable to access those animals in their experiments (63), but Animal Welfare Officers from major research providers have reported that very few animals were culled due to the cancellation of research projects. Research projects that had already commenced were permitted to continue once contingency plans were developed and approved. Non-urgent new projects were deferred. Many researchers were given a 12-month extension by their AEC for animal work that had not commenced.

In some research institutions, animal care staff took on research activities (such as monitoring) rather than have researchers attend, and researchers were taught basic animal husbandry in case the animal care staff were unable to work. In primary and secondary schools with animals, rosters were put in place to care for animals during school closure.

### Companion Animals

In the early stages of the pandemic, there was concern that tight controls on human movement and the closure of borders would discourage people from seeking veterinary assistance, leaving home to care for animals (e.g., horses in agistment), or transporting supplies and animals. In March, some organizations, including Animal Health Australia (AHA), the Australian Veterinary Association (AVA), and the Royal Society for the Prevention of Cruelty to Animals (RSPCA) Australia, contacted the Australian Federal Department of Agriculture, Water and the Environment to request that veterinarians be classified as an essential service. The request was granted. The AVA also re-affirmed that veterinarians could conduct telemedicine consultations in the context of the COVID-19 pandemic (64).

Many animal shelters struggled financially due to the closure of charity shops and fundraising events that normally provide support for their work. At the beginning of the pandemic, RSPCA Australia led a “clear the shelter” campaign, which was highly successful in increasing pet adoptions, and non-RSPCA shelters reported an increase in pet adoptions. It was suggested that the government “stay at home” directions also might have motivated some people to adopt pets (65). There were some delays in pet adoptions, because the gonadectomization of animals was considered a non-essential surgery and was discouraged (although not prohibited) during lockdown (66). By late June, there had been some returns of animals to shelters, but the overall outcome was a net reduction of animals in shelters (67). Veterinarians reported a rise in pet behavioral issues (68), which might have been a result of the increase in adoptions.

Another consideration was how animal care could be sustained if a pet owner became sick or hospitalized. To deal with that risk, some city councils and shelters implemented networks of foster carers (69). There was concern that horse owners might be unwilling or unable to meet the expense of maintaining their horses if the owners lost income. A few cases of COVID-19 in dogs and cats were reported in different countries around the world (70), and there was concern that public fear of zoonotic transmission might cause people to abandon their pets. Several organizations provided information to the public to assert that pets were not considered at risk of contracting or spreading COVID-19 in households and the community (71). RSPCA Australia also provided information on advising pet owners on how to care for animals under the restrictions, how to keep citizens and their animals safe, and also to address potential welfare issues such as lack of socialization of puppies, behavioral and welfare issues when owners went back to work, and animals being scared by people wearing masks. RSPCA Australia also provided emergency resources for people on how to make plans for their animals in the case that they might get sick or be unable to care for their animals, either during the pandemic or for other reasons.

## Formation of a National, Multisectoral Response Group

On 24 March 2020, the Zoo and Aquarium Association approached The Animal Welfare Collaborative (TAWC), a collective of universities, organizations, companies, and individuals that was formed in 2018 with the common goal of improving the welfare of animals. ZAA sought information on how other animal sectors were responding to COVID-19-related challenges. TAWC responded by contacting the Australian Federal Department of Agriculture, Water and the Environment; Animal Health Australia; and the National Primary Industries Animal Welfare Research, Development and Extension Strategy (NAWRDES). On 27 March, TAWC, NAWRDES, and AHA created a national, multisectoral response group, called the COVID-19 Animal Welfare Response Reference Group (COVAWRRG). More than 34 organizations participated in the group, including the Federal, State and Territory departments of primary industries, animal protection organizations, the livestock production and processing sectors, the aquaculture, wildlife, and animal racing sectors, the zoo and aquarium sector, the animals in research and teaching sector, and the companion animal sector. The COVAWRRG met weekly during the early stages of the pandemic, and later fortnightly, to share critical sector updates on emerging COVID-19-related challenges to animal welfare, and to coordinate responses.

Within the first 2 months of the pandemic, key outcomes of the COVAWRRG included clarification on the regulation of the cross-border transport of animals, identification of the need to travel across state and intrastate borders to care for animals, and considerations for rapid-depopulation and culling in response to COVID-19 impacts on processing capacity. The need to classify veterinarians and other key personnel as an essential service, and to support zoos, aquariums, and wildlife parks in financial hardship were also key issues raised in the COVAWRRG. During the second wave of COVID-19, government statements and industry plans addressed animal welfare concerns with greater clarity than they did during the first wave, a preparedness that may have resulted in part from COVAWRRG discussions. For example, the statement by the Premier of Victoria on 3 August 2020 that indicated that Stage 4 restrictions were to be reinstated, specified that all agricultural, food production businesses, animal care, and necessary support services could continue to operate as normal. The emphasis on animal care could be attributed to the awareness of the issues that had been raised.

## Recommendations for Future Crises

1. **National risk assessment –** There is a need for a national framework for risk assessment of animal welfare, across all sectors, to identify potential risks to animal welfare, not just animal disease risks.
2. **Clear communication channels –** There is a need for clear communication channels among industries and Organizations that deal with animals and their welfare, and for consistent, streamlined, and easy-to-access communication strategies for the public.
3. **Contingency plans for animal welfare –** Sectors that are responsible for the care of animals need crisis response plans in place, covering everything that could disrupt animal care. Risks include natural disasters, biosecurity events, supply chain shocks, Labor disruptions, movement restrictions on personnel, financial hardship, feed supply shortage, and limitations to transport and processing capacity. Contingency plans must cover financial hardship, and they need to identify resource reserves that can be used to ensure that the care of the animals is not compromised. Sectors should document the arrangements that were developed for the COVID-19 pandemic and incorporate these into contingency plans.
4. **Crisis response group** – In the event of a crisis that has not been forecasted, it is essential to quickly assemble relevant sectors to identify common issues, coordinate responses, exchange information and support, and develop appropriate solutions. The COVAWRRG might have been the first example of such a cross-sectoral crisis response group in animal welfare. Ongoing collaborative partnerships among sectors will no doubt facilitate the assembly of a crisis response group in the future.
5. **Support systems for animal care providers –** Challenges to human mental health during a crisis may compromise one's ability to provide adequate care for animals or to perceive when animal welfare is at risk. Support systems for animal care providers during times of crisis should be developed by each sector.

## Conclusion

The COVID-19 pandemic created angst and fear across society in addition to the health threats that it posed and touched all Australian sectors that care for animals. The spontaneous formation of the COVAWRRG, a national, multisectoral response group on animal welfare, facilitated the flow of information and helped to mitigate shocks to different sectors and to ensure the continuity of care for animals. The COVAWRRG provided a platform for communication and collaboration for 34 diverse organizations, including the Federal, State and Territory departments of primary industries, animal protection organizations, the livestock production and processing sectors, the aquaculture, wildlife, and animal racing sectors, the zoo and aquarium sector, the animals in research and teaching sector, and the companion animal sector. The activity of the COVAWRRG demonstrated that the responsibility for animal welfare is shared by multiple enactors across society, and therefore, multisectoral collaboration is an efficient way of addressing complex challenges in animal welfare (72). The experience of the COVAWRRG provides insight on mechanisms to ensure that animal welfare is not compromised during unpredictable events, such as the COVID-19 pandemic.

## Data Availability Statement

The original contributions generated for this study are included in the article/supplementary material, further inquiries can be directed to the corresponding author/s.

## Author Contributions

DB, JF, SM, and AT drafted the manuscript. All authors provided information and contributed to the editing of the manuscript.

## Conflict of Interest

JB was employed by the company JB Consulting and author BP was employed by the company Australian Wool Innovation Ltd. The remaining authors declare that the research was conducted in the absence of any commercial or financial relationships that could be construed as a potential conflict of interest.
